# Supplementary material for: Admissions to psychiatric inpatient services and use of coercive measures in 2020 in a Swiss psychiatric department: An interrupted time-series analysis
Source: PLoS One. 2023 Jul 27;18(7):e0289310. doi: 10.1371/journal.pone.0289310 (PMC10374153; doi:10.1371/journal.pone.0289310)
Supplement: S1 Appendix — (DOCX) [file pone.0289310.s001.docx]

**S1 Appendix. Study variables by year**

|  | **Observed values** | | | **Predicted values** | |
| --- | --- | --- | --- | --- | --- |
| **Time** | **No. hospital.** | **% coercive** | **HoNOS** | **% coercive** | **95% CI** |
| 2019w01 | 256 | 62.9 | 20.1 | . | . |
| 2019w02 | 287 | 37.3 | 21.4 | 56.4 | 40.8, 72.0 |
| 2019w03 | 301 | 42.9 | 23.5 | 42.5 | 28.4, 56.6 |
| 2019w04 | 295 | 42.4 | 25.8 | 43.8 | 30.0, 57.5 |
| 2019w05 | 304 | 41.1 | 21.4 | 41.2 | 27.6, 54.8 |
| 2019w06 | 292 | 37.3 | 23.1 | 43.5 | 29.9, 57.1 |
| 2019w07 | 290 | 40.7 | 24.2 | 41.1 | 27.5, 54.7 |
| 2019w08 | 300 | 55.3 | 23.3 | 38.8 | 25.2, 52.4 |
| 2019w09 | 298 | 29.2 | 22.8 | 46.3 | 32.7, 59.9 |
| 2019w10 | 288 | 46.9 | 22.8 | 40.8 | 27.2, 54.4 |
| 2019w11 | 301 | 44.2 | 22.3 | 40.7 | 27.1, 54.3 |
| 2019w12 | 291 | 41.6 | 25.7 | 44.1 | 30.6, 57.7 |
| 2019w13 | 296 | 39.2 | 22.8 | 41.9 | 28.3, 55.5 |
| 2019w14 | 304 | 56.9 | 23.9 | 39.0 | 25.4, 52.6 |
| 2019w15 | 297 | 37.7 | 23.3 | 48.1 | 34.5, 61.7 |
| 2019w16 | 305 | 41.6 | 24.2 | 41.8 | 28.2, 55.4 |
| 2019w17 | 285 | 41.4 | 23.1 | 45.8 | 32.2, 59.3 |
| 2019w18 | 290 | 31.7 | 25.6 | 42.7 | 29.1, 56.3 |
| 2019w19 | 294 | 28.9 | 24.2 | 37.0 | 23.4, 50.6 |
| 2019w20 | 286 | 30.4 | 23.1 | 35.0 | 21.4, 48.6 |
| 2019w21 | 279 | 31.9 | 25.4 | 34.4 | 20.8, 47.9 |
| 2019w22 | 275 | 28.0 | 22.5 | 34.0 | 20.4, 47.6 |
| 2019w23 | 282 | 23.8 | 22.4 | 29.8 | 16.2, 43.4 |
| 2019w24 | 271 | 13.7 | 25.0 | 29.3 | 15.8, 42.9 |
| 2019w25 | 280 | 17.1 | 23.4 | 20.6 | 07.0, 34.2 |
| 2019w26 | 282 | 21.6 | 26.1 | 18.6 | 05.0, 32.1 |
| 2019w27 | 271 | 24.4 | 24.6 | 22.0 | 08.5, 35.6 |
| 2019w28 | 279 | 15.8 | 21.6 | 21.3 | 07.7, 34.9 |
| 2019w29 | 275 | 29.1 | 21.5 | 19.6 | 06.0, 33.2 |
| 2019w30 | 288 | 24.0 | 22.7 | 21.0 | 07.4, 34.5 |
| 2019w31 | 281 | 21.0 | 23.9 | 23.6 | 10.0, 37.2 |
| 2019w32 | 289 | 24.6 | 23.4 | 20.7 | 07.1, 34.3 |
| 2019w33 | 295 | 30.5 | 22.4 | 21.0 | 07.5, 34.6 |
| 2019w34 | 293 | 23.2 | 23.3 | 25.5 | 11.9, 39.1 |
| 2019w35 | 295 | 32.2 | 23.9 | 24.0 | 10.4, 37.6 |
| 2019w36 | 308 | 36.7 | 23.3 | 24.8 | 11.2, 38.4 |
| 2019w37 | 303 | 39.6 | 25.5 | 30.9 | 17.3, 44.5 |
| 2019w38 | 296 | 39.9 | 24.0 | 36.0 | 22.4, 49.6 |
| 2019w39 | 299 | 24.7 | 24.9 | 37.0 | 23.4, 50.6 |
| 2019w40 | 305 | 20.0 | 24.0 | 30.3 | 16.7, 43.9 |
| 2019w41 | 315 | 18.4 | 21.9 | 23.7 | 10.1, 37.2 |
| 2019w42 | 314 | 22.3 | 24.3 | 21.5 | 07.9, 35.1 |
| 2019w43 | 317 | 25.2 | 25.1 | 21.1 | 07.5, 34.7 |
| 2019w44 | 293 | 27.3 | 24.6 | 27.7 | 14.1, 41.3 |
| 2019w45 | 297 | 23.2 | 24.6 | 26.6 | 13.0, 40.2 |
| 2019w46 | 302 | 23.8 | 24.0 | 24.0 | 10.4, 37.6 |
| 2019w47 | 304 | 22.7 | 22.7 | 23.4 | 09.8, 37.0 |
| 2019w48 | 304 | 20.1 | 23.5 | 23.0 | 09.4, 36.6 |
| 2019w49 | 303 | 28.4 | 22.9 | 21.8 | 08.2, 35.4 |
| 2019w50 | 303 | 33.0 | 24.8 | 24.6 | 11.0, 38.2 |
| 2019w51 | 317 | 29.7 | 23.4 | 25.3 | 11.7, 38.8 |
| 2019w52 | 288 | 39.6 | 22.7 | 33.0 | 19.4, 46.6 |
| 2020w01 | 287 | 33.1 | 23.2 | 36.0 | 22.4, 49.6 |
| 2020w02 | 296 | 38.2 | 22.0 | 32.8 | 19.2, 46.4 |
| 2020w03 | 305 | 31.5 | 22.6 | 33.2 | 19.6, 46.8 |
| 2020w04 | 305 | 30.5 | 22.8 | 32.3 | 18.7, 45.9 |
| 2020w05 | 321 | 30.2 | 22.5 | 28.1 | 14.5, 41.7 |
| 2020w06 | 314 | 26.1 | 23.1 | 30.4 | 16.8, 44.0 |
| 2020w07 | 302 | 30.8 | 23.0 | 30.9 | 17.3, 44.5 |
| 2020w08 | 312 | 34.6 | 23.4 | 28.7 | 15.1, 42.3 |
| 2020w09 | 297 | 25.6 | 22.8 | 34.2 | 20.6, 47.8 |
| 2020w10 | 278 | 31.7 | 24.1 | 34.3 | 20.7, 47.9 |
| 2020w11 | 289 | 34.9 | 22.1 | 30.8 | 17.2, 44.4 |
| 2020w12 | 260 | 41.2 | 24.8 | 38.4 | 24.9, 52.0 |
| 2020w13 | 224 | 41.1 | 26.5 | 46.9 | 33.3, 60.5 |
| 2020w14 | 230 | 50.4 | 24.6 | 43.0 | 29.4, 56.6 |
| 2020w15 | 235 | 55.3 | 24.8 | 45.1 | 31.5, 58.7 |
| 2020w16 | 234 | 54.3 | 22.3 | 49.7 | 36.1, 63.2 |
| 2020w17 | 250 | 40.0 | 24.4 | 48.3 | 34.7, 61.9 |
| 2020w18 | 258 | 41.9 | 23.4 | 42.9 | 29.3, 56.5 |
| 2020w19 | 255 | 42.7 | 25.1 | 43.0 | 29.4, 56.6 |
| 2020w20 | 256 | 45.3 | 25.4 | 42.6 | 29.0, 56.1 |
| 2020w21 | 248 | 46.0 | 23.7 | 45.3 | 31.7, 58.9 |
| 2020w22 | 248 | 44.4 | 24.9 | 45.5 | 31.9, 59.1 |
| 2020w23 | 242 | 41.3 | 25.1 | 46.1 | 32.5, 59.7 |
| 2020w24 | 255 | 44.3 | 24.1 | 41.2 | 27.7, 54.8 |
| 2020w25 | 262 | 47.7 | 26.1 | 41.0 | 27.4, 54.6 |
| 2020w26 | 268 | 35.4 | 25.6 | 42.6 | 29.0, 56.2 |
| 2020w27 | 280 | 42.1 | 24.3 | 36.9 | 23.3, 50.5 |
| 2020w28 | 269 | 48.3 | 23.2 | 41.3 | 27.8, 54.9 |
| 2020w29 | 269 | 49.1 | 24.8 | 44.3 | 30.7, 57.9 |
| 2020w30 | 263 | 51.7 | 23.4 | 47.5 | 33.9, 61.1 |
| 2020w31 | 256 | 48.0 | 24.8 | 50.7 | 37.1, 64.3 |
| 2020w32 | 259 | 49.4 | 25.2 | 48.8 | 35.2, 62.4 |
| 2020w33 | 255 | 57.3 | 25.0 | 49.8 | 36.2, 63.4 |
| 2020w34 | 248 | 50.0 | 24.8 | 54.4 | 40.8, 68.0 |
| 2020w35 | 237 | 48.5 | 25.1 | 54.6 | 41.0, 68.2 |
| 2020w36 | 240 | 44.2 | 25.2 | 51.2 | 37.7, 64.8 |
| 2020w37 | 242 | 45.5 | 23.4 | 47.6 | 34.1, 61.2 |
| 2020w38 | 253 | 36.0 | 23.9 | 44.3 | 30.7, 57.9 |
| 2020w39 | 265 | 33.2 | 24.7 | 38.1 | 24.5, 51.7 |
| 2020w40 | 264 | 43.6 | 24.0 | 36.1 | 22.5, 49.7 |
| 2020w41 | 262 | 41.2 | 24.4 | 39.6 | 26.0, 53.2 |
| 2020w42 | 260 | 29.2 | 23.7 | 40.6 | 27.0, 54.2 |
| 2020w43 | 255 | 36.9 | 22.6 | 36.6 | 23.0, 50.2 |
| 2020w44 | 233 | 50.2 | 24.6 | 41.1 | 27.5, 54.7 |
| 2020w45 | 225 | 44.0 | 21.6 | 46.6 | 33.0, 60.2 |
| 2020w46 | 215 | 60.5 | 24.3 | 47.4 | 33.8, 61.0 |
| 2020w47 | 230 | 56.1 | 21.2 | 49.9 | 36.3, 63.5 |
| 2020w48 | 242 | 33.5 | 22.2 | 50.0 | 36.4, 63.6 |
| 2020w49 | 239 | 42.3 | 22.0 | 43.3 | 29.7, 56.9 |
| 2020w50 | 249 | 43.4 | 22.7 | 40.7 | 27.1, 54.3 |
| 2020w51 | 244 | 54.9 | 21.1 | 42.8 | 29.2, 56.4 |
| 2020w52 | 248 | 42.3 | 19.3 | 47.1 | 33.5, 60.7 |

w = week, No. hospital. = Number of hospitalizations, HoNOS = Health of Nations Outcome Scale, CI= confidence interval.
