# Supplementary material for: Admissions to psychiatric inpatient services and use of coercive measures in 2020 in a Swiss psychiatric department: An interrupted time-series analysis
Source: PLoS One. 2023 Jul 27;18(7):e0289310. doi: 10.1371/journal.pone.0289310 (PMC10374153; doi:10.1371/journal.pone.0289310)
Supplement: Attachment — Submitted filename: Response_Wullschleger_20230711.docx [file pone.0289310.s005.docx]

**Cover letter for the revised manuscript:**

“Admissions to psychiatric inpatient services and use of coercive measures in 2020 in a Swiss psychiatric Department: an interrupted time-series analysis”

Dear Prof. Gulsham,

We thank you for the opportunity you gave us to submit again a revised version of our manuscript.

Point by point response:

**Additional Editor Comments:**

**1. Move table 1 to appendix.**

Table 1 has been moved to appendix.

**2. A thorough grammar check is necessary.**

The manuscript has been thoroughly checked regarding language and grammar.

We sincerely hope that this new version will now fully comply with the publication standards of PLOS One. We are looking forward to your feedback.

Best regards,

For the research team

Alexandre Wullschleger
